# Pathogenicity comparison between QX-type and Mass-type infectious bronchitis virus to different segments of the oviducts in laying phase

**DOI:** 10.1186/s12985-022-01788-0

**Published:** 2022-04-07

**Authors:** Xiaorong Zhang, Kun Yan, Chengcheng Zhang, Mengjiao Guo, Shuqin Chen, Kai Liao, Zongyi Bo, Yongzhong Cao, Yantao Wu

**Affiliations:** 1 Jiangsu Co-Innovation Center for the Prevention and Control of Animal Infectious Disease and Zoonoses, College of Veterinary Medicine, Yangzhou University, Yangzhou, 225009 Jiangsu China; 2 The International Joint Research Laboratory of Agricultural and Agri-Product Safety, Yangzhou University, Yangzhou, 225009 Jiangsu China

**Keywords:** Infectious bronchitis virus, QX-type, Pathogenicity, Oviduct, Laying phase, Egg quality

## Abstract

**Background:**

The QX-type infectious bronchitis virus (IBV) has become the predominant genotype worldwide in recent years and has caused serious economic losses to the chicken industry. The most significant feature of QX IBV is that its infection in the early growing stage can cause abnormal oviduct development, resulting in a high proportion of ‘false layers’ in poultry flocks of laying hens and breeders. However, few studies have evaluated whether infections of QX-type IBV in laying stages can also cause severe pathological changes in the oviduct.

**Methods:**

In this study, 300-day-old specific-pathogen-free chickens were infected either with the QX-type strain QXL or Massachusetts (Mass)-type strain M41 to compare their pathogenicity on different segments of the oviduct.

**Results:**

Both the QXL and M41 strains successfully replicated in all segments of the oviduct; however, the QXL strain was more highly distributed in mucosal layer and caused severe lesions in the lamina propria, including interstitial dilation, inflammatory cell infiltration, and distinct expansion of tubular glands. Moreover, the QXL strain induced high expression of proinflammatory cytokines and cytotoxic molecules in the majority of segments in the oviduct. Further research found that the QXL strain may affected the formation of shell membranes and eggshells by inhibiting the expression of type I collagen and CaBP-D28k.

**Conclusions:**

Our results indicate that the QX-type IBV is more pathogenic than Mass-type IBV to oviduct in laying phase. Collectively, these findings provide detailed information on the pathological changes in different segments of the oviduct in laying phase, which could offer a better understanding about the pathogenicity of IBV.

**Supplementary Information:**

The online version contains supplementary material available at 10.1186/s12985-022-01788-0.

## Background

Avian infectious bronchitis (IB) is a highly contagious, acute viral respiratory disease caused by IB virus (IBV), which primarily infects chickens [1]. As a respiratory disease virus, IBV initially targets the epithelium of the bronchus and induces acute respiratory symptoms, such as tracheal rales and dyspnea [2]. Some strains can also spread to the urogenital system, causing severe nephritis and reduced egg production and quality in layer hens. Furthermore, infected chickens are also susceptible to secondary infections with mycoplasma, bacteria, or other pathogens, leading to increased mortality [3].

In China, QX-type IBV was first reported to be isolated from a broiler chicken flocks that had proventriculitis in 1997 in Qingdao, Shandong province [4], and in the past two decades, outbreaks have also been found in many countries and regions around the world, and gradually developed into the prevailing genotype in many areas [5–7]. QX-type IBV is highly pathogenic to multiple organs, which can cause severe tracheal cilia injury, noticeably swollen pale kidneys, abnormal development of the oviduct and ovarian follicles [8, 9]. Infected chickens often manifest characteristic dilatation and serum-like fluid accumulation in the oviduct, which is known as the cystic oviduct [10, 11]. During the laying period, although the ovaries of infected chickens develop normally, the vitellus falls into the abdominal cavity, which causes peritonitis due to degeneration and shutting of the oviduct. A condition called ‘false layers’ syndrome develops in this course. The severity of reproductive disease varies greatly depending on the virus strain, period of infection and individual host conditions [12]. Different strains belonging to the QX type also demonstrated significant differences in pathogenicity [13]. Although it is known that QX-type IBV causes severe damage to the oviduct in young chickens and subsequently disrupts egg laying, the pathogenicity to the oviduct when infection occurs in laying phase is still not well understood. In addition, the oviduct is morphologically divided into four different functional segments, including the infundibulum, magnum, isthmus and uterus. Albumen, eggshell membrane, and eggshell are formed in the magnum, isthmus and uterus, respectively [14]. The eggshell membrane shows fibrous meshwork structures mainly composed of collagens and glycoprotein [15]. Types I and V collagen exist in egg shell membrane and the ratio of these is approximately 100:1 [16]. In the avian eggshell gland, calcium-binding protein (calbindin, CaBP) exists as a high-molecular-weight protein of 28 kDa (CaBP-D28k) and plays a role in the transportation of Ca^2+^ for shell formation [17]. Therefore, it is necessary to examine the influence of the QX-type IBV on different segments of the oviduct.

The aim of this study was to provide a better understanding of the pathogenicity of IBV. To this end, the QX-type strain QXL and the Mass-type strain M41 (primarily causing respiratory symptoms) were used to investigate and compare pathogenicity in different segments of the oviduct. Pathological changes were evaluated by examining the antigen distribution, lesions, and expression of proinflammatory cytokines, cytotoxic molecules, type I interferon and eggshell formation-related genes.

## Materials and methods

### Animals

SPF embryonated chicken eggs were purchased from Beijing Boehringer Ingelheim Vital Biotechnology Co., Ltd. (Beijing, China). SPF white leghorn chickens were purchased from Jinan Sipai Furui Livestock Technology Co., Ltd. (Shandong, China).

### Viruses

The QX-type IBV strain CK/CH/JS/2010/12 (abbreviation: QXL) was isolated in 2010 from the trachea and kidney of chickens in a broiler flock exhibiting respiratory signs and death [18]. Mass-type IBV strain M41 was purchased from the China Veterinary Culture Collection Center (CVCC AV1511). QXL and M41 strains were serially diluted (10^–1^–10^–8^), five replicate samples of each dilution were inoculated into 10-day-old SPF embryonated chicken eggs, and the 50% chicken embryo infectious dose (EID_50_) was calculated using the method of Reed and Muench [19].

### Experimental design

A total of 18 leghorn white chickens were kept in negative-pressure isolators under 12 h of light and provided with sufficient amount of food and water until they reached 300 days of age. They were then randomly divided into three groups, two challenge and one control groups (n = 6 each). Birds in two challenge groups were inoculated via the oculonasal route with 200 μL of allantoic fluid containing 10^4^ EID_50_ of IBV strains QXL and M41, one group per strain. Birds in the control group were administered 200 μL of saline solution via the oculonasal route. On days 4 and 8 post inoculation, three animals from each group were randomly selected and euthanized by cervical dislocation to evaluate macroscopic lesions in the oviduct. Tissue samples from the same regions of the infundibulum, magnum, isthmus and uterus of each bird were collected (Additional file 1).

### Histopathology and immunohistochemistry

The four segments of the oviduct were fixed in 10% neutral-buffered formalin, processed via the standard histological procedure, embedded in paraffin wax, and cut into 4 μm sections. Some of the paraffin sections were stained with Hansen’s hematoxylin and eosin for histological observation, whereas other sections were prepared for immunohistochemistry (IHC) to detect the intensity of the viral antigen. Briefly, sections were incubated with 10% (v/v) goat serum (blocking solution) for 30 min. They were then incubated with a 1:15,000 dilution of mouse monoclonal antibody to IBV N protein at 37 ℃ for 2 h [20]. After washing the sections with PBS (3 × 5 min), the immunoreaction products were detected using a concentrated SABC-POD (mouse IgG) kit (BOSTER Bioengineering Co. Ltd., catalog #SA2001). Sections were visualized using DAB and counterstained with Hansen’s hematoxylin. All sections were observed using an optical microscope (Leica, Wetzlar, Germany).

### RT-qPCR analysis of the expression of genes related to the immune response and eggshell formation

Total RNA from different segments of the oviduct (100 mg tissue from each segment) was extracted using an Ultrapure RNA Kit (CoWin Biosciences Co. Ltd., catalog #CW0581M). The concentration of RNA in each sample was measured using an Ultra micro sample spectrophotometer (Thermo, USA), and then 1 μg RNA was reverse transcribed using an EasyScript® Reverse Transcriptase (M-MLV, RNaseH-) Kit (TransGen Biotech Co. Ltd., catalog #N10227) and Oligo (dT)_18_ (Takara Bio, catalog #3806) according to the manufacturer’s instructions. The reaction was performed at 42 °C for 30 min and at 85 °C for 5 s for cDNA synthesis. The RT-qPCR mixture was composed of 10 μL AceQ® qPCR SYBR Green Master Mix (Vazyme Biotech Co. Ltd., catalog #Q111-02/03), 0.4 μL of forward primer, 0.4 μL of reverse primer, 7.2 μL of nuclease-free water and 2 μL cDNA (or nuclease-free water for the control). The thermal profile for RT-qPCR was 95 °C for 5 min, followed by 40 cycles of 95 °C for 10 s, 60 °C for 30 s, melting at 95 °C for 15 s, 60 °C for 60 s and 95 °C for 15 s using a LineGene 9600 Plus (Bioer Technology Co. Ltd., Hangzhou, China). The primers used for RT-qPCR were described previously [21, 22]. The target genes were expressed as a ratio relative to the RPS17 housekeeping gene, and the values were calculated using the 2^−ΔΔCT^ method [23].

### Statistical analysis

GraphPad Prism 8.3 software (GraphPad, La Jolla, CA, USA) was used to analyze all statistical data via one-way analysis of variance. Differences between the challenge and control groups were considered statistically significant when the P-value was < 0.05, highly significant at *P* < 0.01, and extremely significant at *P* < 0.0001.

## Results

### Histopathology and immunohistochemistry

**Fig. 1:**
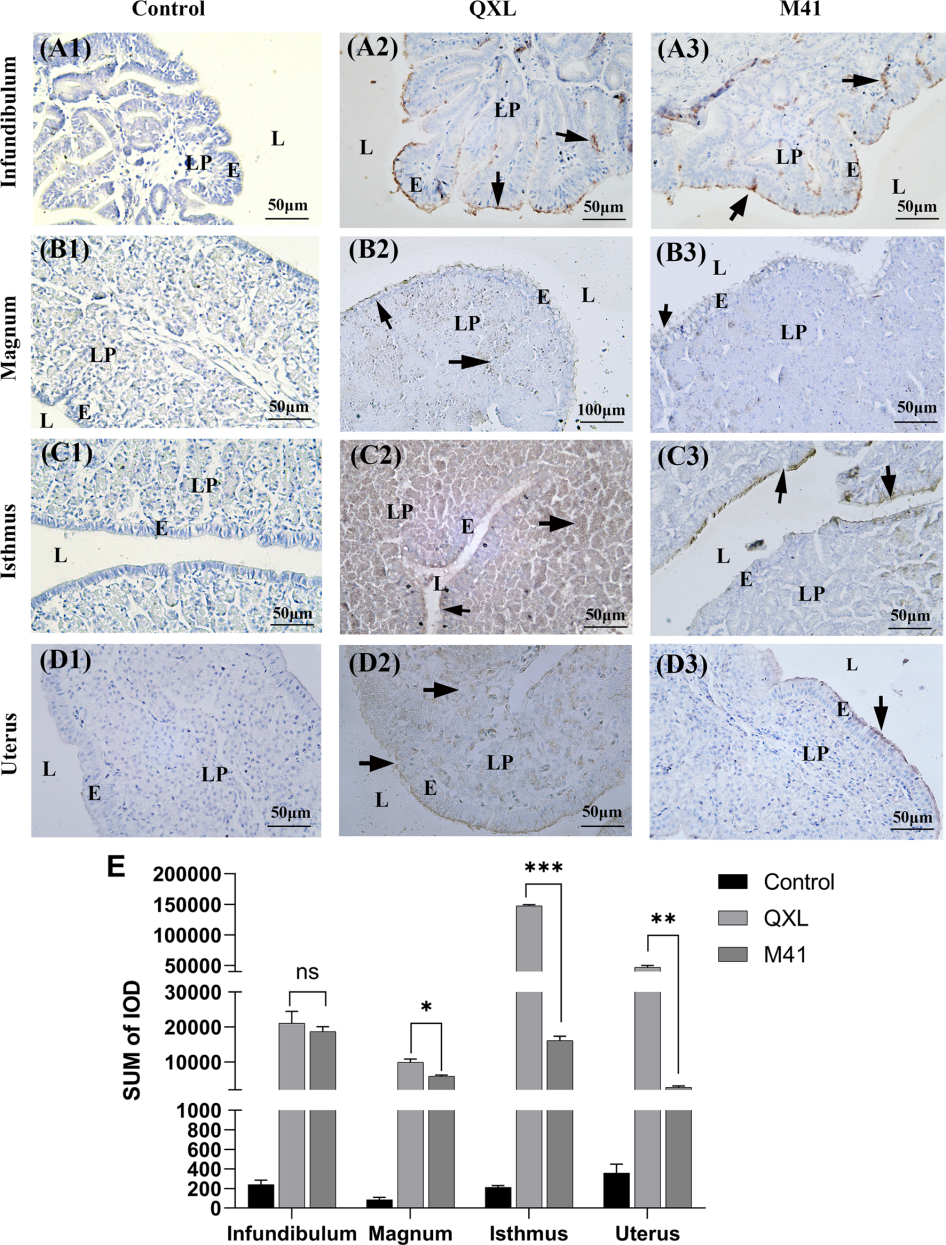
Immunohistochemistry detection of QX and Mass-type IBV antigens 4 days post infection. **A1–D1** Different segments of oviduct in the control group. **A2–D2** Different segments of oviduct in the QXL group. **A3–D3** Different segments of oviduct in the M41 group. **E** The sum of IOD in different segments of oviduct. *P < 0.05, **P < 0.01, ***P < 0.001 represent significant differences between the QXL group and the M41 group. Black arrows indicate antigen deposition. Scale bars: 50 or 100 μm. E, mucosal epithelium; L, lumen; LP, lamina propria

To evaluate the distribution of viral antigen in different segments of oviduct, the paraffin sections were stained with IHC method. The results of IHC showed that the presence of QXL strain antigen was widely detected in the mucosal epithelial cilia and lamina propria (Fig. 1 A2, B2, C2, D2), whereas that of the M41 strain antigen was primarily detected in the mucosal epithelial cilia (Fig. 1 A3, B3, C3, D3). The distribution of the QXL strain antigen in the isthmus was more diffuse than that in the other segments of the oviduct (Fig. 1 C2). The integrated optical density (IOD) of QXL group was significantly higher than M41 group in the magnum, isthmus and uterus (Fig. 1E).

**Fig. 2:**
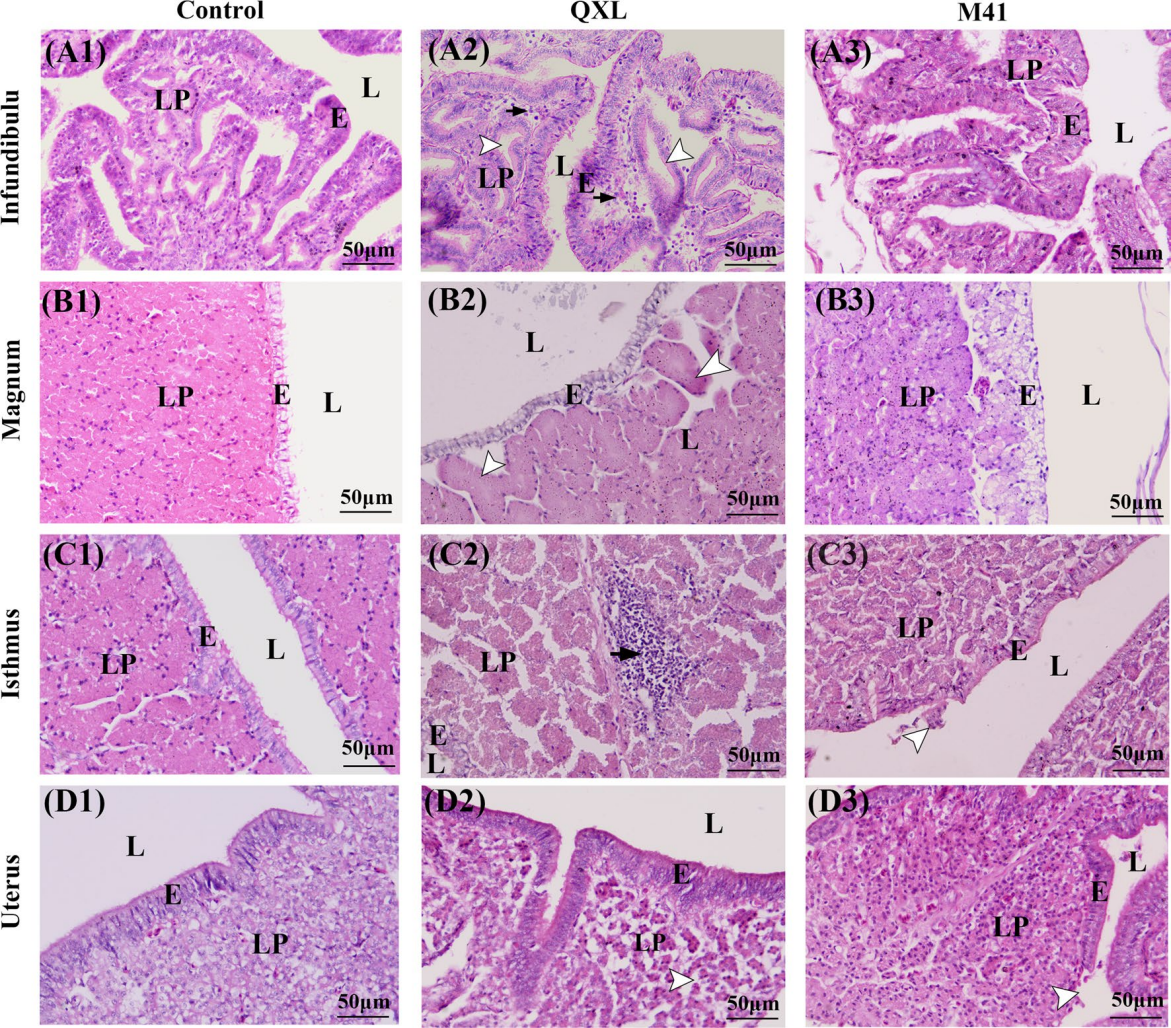
Histopathological analysis of the pathogenicity of QX and Mass-type IBV in the oviduct 4 days post infection. **A1–D1** Different segments of oviduct in the control group. **A2** Infundibulum of the QXL group, black arrows indicate macrophages and lymphocytes distributed in the connective tissue in the lamina propria, white arrows indicate the expansion of tubular glands. **B2** Magnum of the QXL group, white arrows indicate the expansion of tubular glands. **C2** Isthmus of the QXL group, black arrows indicate lymphocytes infiltrating the interstitium of the lamina propria. **D2** Uterus of the QXL group, white arrows indicate interstitial dilation. **C3** Isthmus of the M41 group, white arrows indicate the desquamation of epithelial cells. **D3** Uterus of the M41 group, white arrows indicate the desquamation of epithelial cells. Scale bars: 50 μm. E, mucosal epithelium; L, lumen; LP, lamina propria

The H.E staining method was used to evaluate the pathological changes in different segments of oviduct. In the control group, the mucosal epithelium of the infundibulum, magnum, isthmus and uterus was lined by a ciliated pseudostratified epithelium, and the lamina propria was filled with tubular glands (Fig. 2 A1, B1, C1, D1). In the QXL group, many lymphocytes had infiltrated into the connective tissue of the lamina propria (Fig. 2 A2, C2), whereas inflammatory cells were rarely observed in the M41 group. Distinct expansion of tubular glands was observed in the infundibulum and magnum of the QXL group (Fig. 2 A2, B2), whereas desquamation of epithelial cells was observed in the isthmus and uterus of the M41 group (Fig. 2 C3, D2). Furthermore, the density of tubular glands was decreased due to interstitial dilation of the lamina propria in the uterus of the QXL group (Fig. 2 D2).

### Changes in the expression of proinflammatory cytokine genes and cytotoxic immunoreaction-related genes

**Fig. 3:**
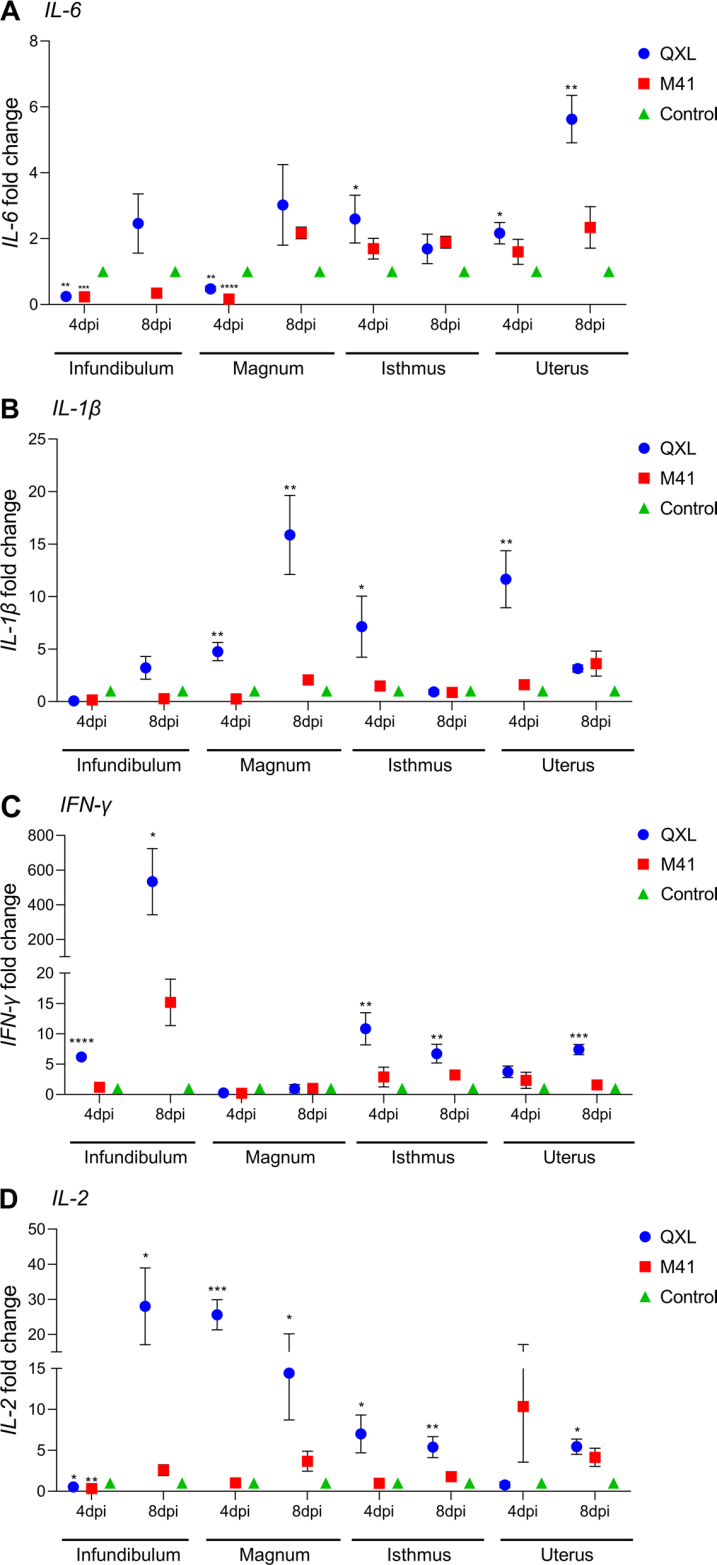
Effects of QX and Mass-type IBV in different segments of the oviduct on mRNA expression of proinflammatory cytokine genes. **A** IL-6, **B** IL-1β, **C** IFN-γ and **D** IL-2. Values are shown as the mean ± SEM (n = 3) of fold change in expression. *P < 0.05, **P < 0.01, ***P < 0.001, ****P < 0.0001 represent significant differences between the infection groups and the control group. DPI, days post infection; SEM, standard error of the mean

To evaluate the degree of inflammation in each segment of the oviduct, the expression of proinflammatory cytokines (IL-6, IL-1β, IFN-γ, and IL-2) was detected 4 and 8 days after infection. Expression of IL-6 was significantly higher in the QXL group than in the control group in the isthmus at 4 days and in the uterus 4 and 8 days after infection, whereas its expression was significantly lower in both the QXL and M41 groups than in the control group in the infundibulum and magnum at 4 days (Fig. 3A). The expression of IL-1β was significantly higher in the QXL group than in the control group in the magnum at 4 and 8 days, and in the isthmus and uterus at 4 days (Fig. 3B). The expression of IFN-γ was significantly higher in the QXL group than in the control group in the infundibulum and isthmus at 4 and 8 days, which was also in the uterus at 8 days (Fig. 3C). IL-2 expression was significantly higher in the QXL group than in the control group in the infundibulum and uterus at 8 days, and in the magnum and isthmus at 4 and 8 days (Fig. 3D).

**Fig. 4:**
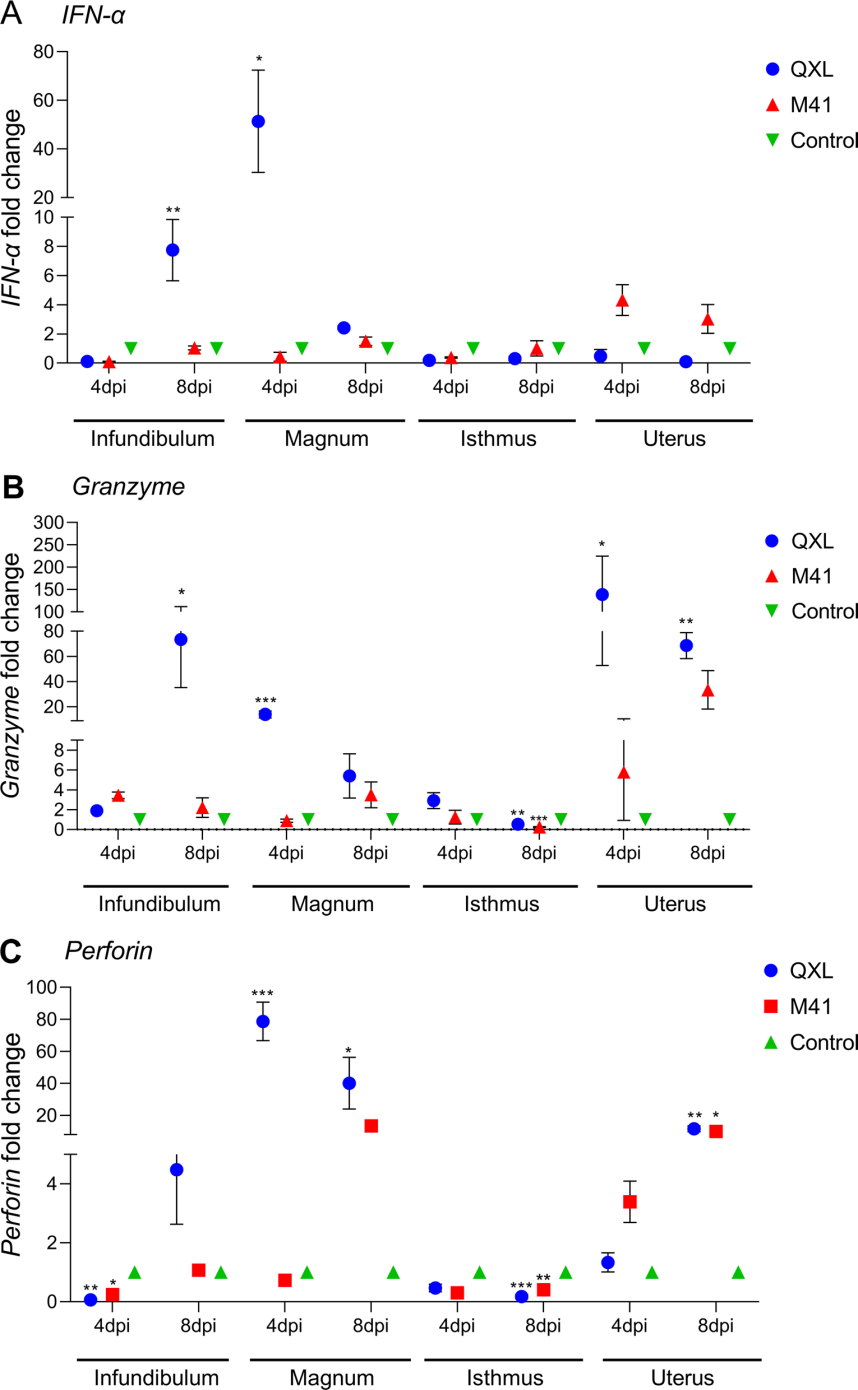
Effects of QX and Mass-type IBV in different segments of the oviduct on mRNA expression of IFN-α (**A**), granzyme (**B**) and perforin (**C**). Values are shown as the mean ± SEM (n = 3) of fold change in the expression. *P < 0.05, **P < 0.01, ***P < 0.001 represent significant differences between the infection groups and the control group. DPI, days post infection; SEM, standard error of the mean

Cytotoxic molecules (granzyme and perforin) and IFN-α were subsequently detected at 4 and 8 days after infection. IFN-α expression was significantly higher in the QXL group than in the control group in the infundibulum at 8 days and in the magnum at 4 days (Fig. 4A). Granzyme expression was significantly higher in the QXL group than in the control group in the infundibulum at 8 days, in the magnum at 4 days, and in the uterus at 4 and 8 days (Fig. 4B). The expression of perforin was significantly higher in the QXL group than in the control group in the magnum at 4 and 8 days, and in the uterus at 8 days (Fig. 4C). Moreover, the expression of granzyme and perforin in the isthmus was significantly lower in the QXL and M41 groups compared to the control group at 4 and 8 days (Fig. 4B, C).

### Changes in the expression of eggshell formation-related genes

**Fig. 5:**
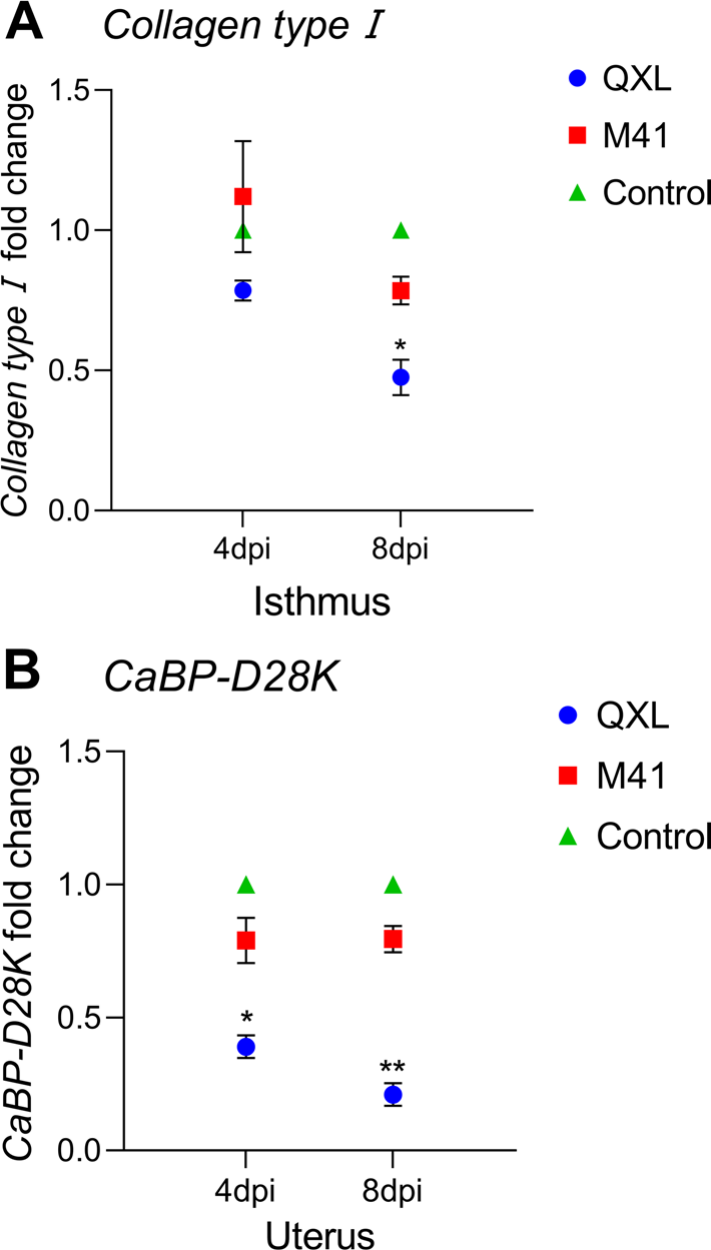
Effects of QX and Mass-type IBV in the isthmus and uterus on the mRNA expression of type I collagen (**A**) and CaBP-D28K (**B**). Values are shown as the mean ± SEM (n = 3) of fold change in the expression. *P < 0.05, **P < 0.01 represent a significant difference between the infection groups and the control group. DPI, days post infection; SEM, standard error of the mean

To evaluate the expression of genes involved in eggshell formation, collagen type I and CaBP-D28K were detected in the isthmus and uterus, respectively. In the isthmus, the expression of type I collagen in the QXL group was significantly lower than that in the control group 8 days after infection (Fig. 5A). The expression of CaBP-D28K in the uterus of the QXL group was significantly lower than that in the control group at 4 and 8 days (Fig. 5B).

## Discussion

Currently, QX is a major genotype of IBV in poultry flocks in Asia, Europe and other parts of the world, causing tremendous economic losses to the poultry industry [24, 25]. Several reports have illustrated the pathogenicity of different QX-type isolates in specific organs of young chickens [26, 27]. However, alterations of the oviduct after viral infection in laying phase are still unknown. In this study, we pioneered the analysis of the pathological effects of QX-type IBV on different segments of the layers’ oviduct and compared them to the Mass-type reference strain M41.

Binding to the host cell is the first crucial step in a virus replication cycle [28]. A previous study confirmed that the QX strain could infect epithelial cells of the pullet’s oviduct in vitro [29]. In this study, we found that both the QXL and M41 strains could infect the epithelial cells of the layers’ oviduct; however, the QXL strain also infected the tubular glands on a large scale. This result suggests that the QXL strain can easily be released from the mucosal epithelium to the lamina propria, possibly via an unknown mechanism mediated by the QXL strain and not by the M41 strain. Previous reports have shown that QX-type IBV caused characteristic dilatation and serum-like fluid accumulation in the oviduct of young chickens, although few alterations were observed microscopically in this organ [10]. In the present study, the QXL strain caused severe injury to different segments of the layers’ oviduct, including the expansion of tubular glands in the infundibulum and magnum, inflammatory cell infiltration in the infundibulum and isthmus, and interstitial dilation of the lamina propria in the uterus. This discrepancy might be caused by the different ages of the birds or by experimental conditions. In the M41 group, only desquamation of epithelial cells was observed in the isthmus and uterus. This result is consistent with a previous study showing that Mass-type IBV causes degeneration and desquamation of epithelial cells in oviduct-organ culture [30].

Generally, the host defends against intracellular viruses via classical inflammatory responses and antiviral immune responses, which are mediated by interferons (IFNs). IFN-γ is a key cytokine for Th1-controlled responses, which is essential for controlling intracellular pathogens [31]. IFN-γ can also cooperate with proinflammatory cytokines, such as IL-1β and IL-6, in the inflammatory response. Previous data demonstrated that infection with attenuated IBV upregulated the expression of proinflammatory cytokines, including IL-1β, IL-6, and IFN-γ [21]. The current results showed that in the QXL group, expression of IL-1β, IL-2, and IL-6 was increased approximately 2–30-fold above normal values, whereas IFN-γ demonstrated a much stronger increase, in some cases more than 500-fold above normal. In this study, macrophages were observed in the infundibulum of the oviduct in the QXL group, where the expression of IFN-γ was increased nearly 500-fold. Type I interferon is a hallmark of innate immune responses to pathogens, and can directly or indirectly act on natural kill (NK) cells, CD8^+^ cytotoxic T lymphocytes (CTLs), dendritic cells (DCs), and B cells to regulate adaptive immune responses [32]. The current results demonstrated that in the QXL group, the expression of IFN-α was elevated in the infundibulum and magnum. Furthermore, we also observed that the levels of granzyme and perforin were significantly increased in the oviduct of the QXL group, except for the isthmus. These data indicate that the QXL strain induces cell-mediated inflammatory responses driven by Th1 cells, subsequently recruiting macrophages, NK cells and other innate immune cells to secrete type I IFN and cytotoxic molecules for virus suppression. Excessive immune responses can also cause oviduct lesions. In the isthmus of the QXL group, however, neither IFN-α nor cytotoxic molecules were detected, and IHC results showed that the viral antigens were most widely distributed in the isthmus. These results suggest that the isthmus is more susceptible to the QXL strain than other segments of the oviduct are. In the M41 strain, expression of proinflammatory cytokines and antiviral molecules was not significantly altered. Thus, we speculate that the ability to induce innate immune responses may be related to viral pathogenicity. This was in agreement with a previous study [33].

Infection with IBV can affect the quality of eggs, such as soft shell eggs and sand shell eggs. Therefore, we evaluated the expression of genes involved in eggshell and eggshell membranes formation. Type I collagen is expressed in the isthmus, and the eggshell membrane forms during the passage of eggs through the oviduct [34]. Previous studies have shown that fibrous components are secreted by tubular glands of isthmus [35]. The current results showed that the expression of type I collagen was downregulated in the isthmus 8 days after challenge in the QXL group. Normally, the mRNA levels of CaBP-D28k in the uterus increases during eggshell calcification [36]. Insufficient calcium deposition is a crucial reason result in poor eggshell quality during the process of eggshell formation. Our results demonstrated that the expression of CaBP-D28k was significantly lower in the QXL group than in the control group at both 4 and 8 days after viral challenge. The decline in the synthesis of type I collagen and CaBP-D28K in the isthmus and uterus might be associated with the elevation of immune-related molecules. Therefore, the QXL strain may affect the formation of shell membranes and eggshells by inhibiting the synthesis of type I collagen and CaBP-D28k. These results also support the findings of Nii et al. [21], who showed that attenuated IBV could decrease the expression of type I collagen and CaBP-D28k.

## Conclusions

The findings presented in this study may help improve the knowledge on QX-type IBV pathogenicity during the laying phase of chickens, and develop strategies to interfere with this infection. However, this study is primarily a description of the phenomenon, the pathogenesis of QX-type IBV to oviduct need the further study. Overall, our findings highlight the high pathogenicity of QX-type IBV to different segments of the oviduct in laying phase, which primarily manifests as marked pathological changes in the lamina propria and disordered expression of genes involved in eggshell and eggshell membranes formation.

## Supplementary Information

**Additional file 1.** Anatomic structure of the oviduct. The red dotted box represents the sampling site.

## Data Availability

All data generated or analyzed during this study are included in this published article [and its supplementary information files].

## Abbreviations

IBV: Infectious bronchitis virus
SPF: Specific-pathogen-free
EID_50_: Embryo infectious dose
IHC: Immunohistochemistry
IOD: Integrated optical density
IFNs: Interferons
IL: Interleukin
NK: Natural killer
CTLs: Cytotoxic T lymphocytes
DCs: Dendritic cells
CaBP: Calcium-binding protein

## References

[1] Cook JK, Jackwood M, Jones RC (2012). The long view: 40 years of infectious bronchitis research. Avian Pathol.

[2] Zhang X, Liao K, Chen S, Yan K, Du X, Zhang C (2020). Evaluation of the reproductive system development and egg-laying performance of hens infected with TW I-type infectious bronchitis virus. Vet Res.

[3] Chhabra R, Chantrey J, Ganapathy K (2015). Immune responses to virulent and vaccine strains of infectious bronchitis viruses in chickens. Viral Immunol.

[4] Liu S, Kong X (2004). A new genotype of nephropathogenic infectious bronchitis virus circulating in vaccinated and non-vaccinated flocks in China. Avian Pathol.

[5] Zhao W, Gao M, Xu Q, Xu Y, Zhao Y, Chen Y (2017). Origin and evolution of LX4 genotype infectious bronchitis coronavirus in China. Vet Microbiol.

[6] Lee H, Jeong S, Cho A, Kim K, Kim J, Park D, et al. Genomic analysis of avian infectious bronchitis viruses recently isolated in South Korea reveals multiple introductions of GI-19 Lineage (QX Genotype). Viruses. 2021;13(6). 10.3390/v13061045 PMC8228071 34072981

[7] Valastro V, Holmes E, Britton P, Fusaro A, Jackwood M, Cattoli G (2016). S1 gene-based phylogeny of infectious bronchitis virus: an attempt to harmonize virus classification. Vet Microbiol.

[8] Shao L, Zhao J, Li L, Huang X, Yang H, Cheng J (2020). Pathogenic characteristics of a QX-like infectious bronchitis virus strain SD in chickens exposed at different ages and protective efficacy of combining live homologous and heterologous vaccination. Vet Res.

[9] Zhong Q, Hu YX, Jin JH, Zhao Y, Zhao J, Zhang GZ (2016). Pathogenicity of virulent infectious bronchitis virus isolate YN on hen ovary and oviduct. Vet Microbiol.

[10] Benyeda Z, Mato T, Suveges T, Szabo E, Kardi V, Abonyi-Toth Z (2009). Comparison of the pathogenicity of QX-like, M41 and 793/B infectious bronchitis strains from different pathological conditions. Avian Pathol.

[11] de Wit J, Nieuwenhuisen-van Wilgen J, Hoogkamer A, van de Sande H, Zuidam G, Fabri THF. Induction of cystic oviducts and protection against early challenge with infectious bronchitis virus serotype D388 (genotype QX) by maternally derived antibodies and by early vaccination. Avian Pathol. 2011;40(5):463–71. 10.1080/03079457.2011.599060 21834621

[12] Dolz R, Vergara-Alert J, Perez M, Pujols J, Majo N (2012). New insights on infectious bronchitis virus pathogenesis: characterization of Italy 02 serotype in chicks and adult hens. Vet Microbiol.

[13] Yan S, Liu X, Zhao J, Xu G, Zhao Y, Zhang G (2017). Analysis of antigenicity and pathogenicity reveals major differences among QX-like infectious bronchitis viruses and other serotypes. Vet Microbiol.

[14] Yukinori, Yoshimura, Animesh, Medicine BJAiE, Biology. Female Reproductive System and Immunology. Adv Exp Med Biol. 2017;1001:33. 10.1007/978-981-10-3975-1_3 28980228

[15] Johnson AL, Whittow GC (2000). Reproduction in the female. Avian physiology.

[16] Wong M, Hendrix M, Mark K, Little C, Stern R (1984). Collagen in the egg shell membranes of the hen. Dev Biol.

[17] Bar A (2009). Calcium transport in strongly calcifying laying birds: mechanisms and regulation. Comp Biochem Physiol A Mol Integr Physiol.

[18] Zhang X. Molecular epidemiology of infectious bronchitis virus in China between 2009 and 2011, and development of recombinant vaccine using Marek’s disease virus as vector. 2012; Ph.D. Thesis, Yangzhou University. (in Chinese with English abstract)

[19] Matumoto M (1949). A note on some points of calculation method of LD50 by Reed and Muench. Jpn J Exp Med.

[20] Du X, Zhao J, Guo M, Li M, Zhang C, Wu Y (2021). Preparation and identification of broadspectrum monoclonal antibody against N protein of infectious bronchitis virus. China Poultry.

[21] Nii T, Isobe N, Yoshimura Y (2014). Effects of avian infectious bronchitis virus antigen on eggshell formation and immunoreaction in hen oviduct. Theriogenology.

[22] Villanueva AI, Kulkarni RR, Sharif S (2011). Synthetic double-stranded RNA oligonucleotides are immunostimulatory for chicken spleen cells. Dev Comp Immunol.

[23] Livak KJ, Schmittgen TD (2001). Analysis of relative gene expression data using real-time quantitative PCR and the 2(-Delta Delta C(T)) Method. Methods.

[24] Hong S, Kwon H, Choi K, Kim J (2017). Comparative genomics of QX-like infectious bronchitis viruses in Korea. Arch Virol.

[25] Franzo G, Tucciarone C, Blanco A, Nofrarías M, Biarnés M, Cortey M (2016). Effect of different vaccination strategies on IBV QX population dynamics and clinical outbreaks. Vaccine.

[26] Cheng J, Huo C, Zhao J, Liu T, Li X, Yan S (2018). Pathogenicity differences between QX-like and Mass-type infectious bronchitis viruses. Vet Microbiol.

[27] Li S, Du L, Xia J, Du J, You G, Wen Y (2019). Antigenic and pathogenic characteristics of QX-type avian infectious bronchitis virus strains isolated in Southwestern China. Viruses.

[28] Wickramasinghe IN, de Vries RP, Grone A, de Haan CA, Verheije MH (2011). Binding of avian coronavirus spike proteins to host factors reflects virus tropism and pathogenicity. J Virol.

[29] Mork AK, Hesse M, Abd El Rahman S, Rautenschlein S, Herrler G, Winter C (2014). Differences in the tissue tropism to chicken oviduct epithelial cells between avian coronavirus IBV strains QX and B1648 are not related to the sialic acid binding properties of their spike proteins. Vet Res.

[30] Pradhan H, Mohanty G, Rajya B (1983). Comparative sensitivities of oviduct and tracheal organ cultures and chicken embryo kidney cell cultures to infectious bronchitis virus. Avian Dis.

[31] Kaiser P, Stäheli P, Karel AS, Pete K, Bernd K (2014). Avian cytokines and chemokines. Avian immunology.

[32] Prchal M, Pilz A, Simma O, Lingnau K, Gabain AV, Strobl B (2009). Type I interferons as mediators of immune adjuvants for T- and B cell-dependent acquired immunity. Vaccine.

[33] Chhabra R, Kuchipudi S, Chantrey J, Ganapathy KJV (2016). Pathogenicity and tissue tropism of infectious bronchitis virus is associated with elevated apoptosis and innate immune responses. Virology.

[34] Ohashi H, Okamoto T, Yoshimura Y (2003). Changes in the type I collagen mRNA expression in the isthmus during the passage of egg through the oviduct in Japanese Quail. J Poult Sci.

[35] Draper MH, Davidson MF, Wyburn GM, Johnston HS (1972). The fine structure of the fibrous membrane forming region of the isthmus of the oviduct of Gallus domesticus. Q J Exp Psychol.

[36] Wasserman RH, Smith CA, Smith CM, Brindak ME, Fullmer CS, Krook L (1991). Immunohistochemical localization of a calcium pump and calbindin-D28k in the oviduct of the laying hen. Histochemistry.

