# Supplementary figures and images for: Pathogenicity comparison between QX-type and Mass-type infectious bronchitis virus to different segments of the oviducts in laying phase
Source: Virol J. 2022 Apr 7;19:62. doi: 10.1186/s12985-022-01788-0 (PMC8991805; doi:10.1186/s12985-022-01788-0)

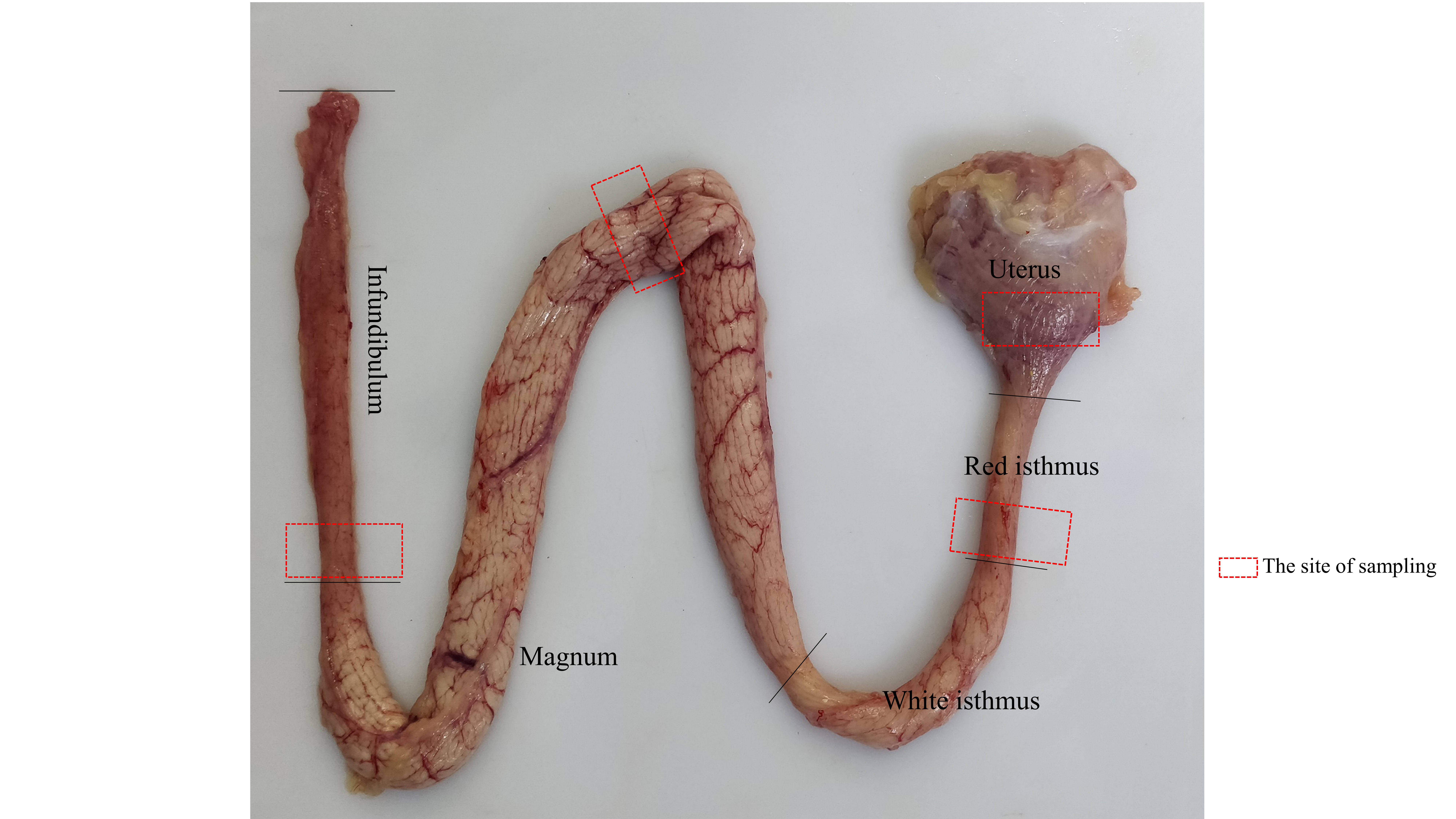

Supplement: Supplementary file 1 — Additional file 1. Anatomic structure of the oviduct. The red dotted box represents the sampling site. [file 12985_2022_1788_MOESM1_ESM.tif]
